# Parenchymal Thoracic Splenosis: History and Nuclear Imaging Without Invasive Procedures May Provide Diagnosis

**DOI:** 10.4021/jocmr401w

**Published:** 2010-07-22

**Authors:** Umer Feroze Malik, Mersadies R. Martin, Rupal Patel, Ahmed Mahmoud

**Affiliations:** a Department of General Internal Medicine, Stanford University Medical Center, Stanford, California, USA; b Department of Surgery, Michigan State University, USA; c Department of Pediatrics at University of Oklahoma, USA; d Department of Surgery, San Joaquin General Hospital, California, USA

## Abstract

Splenosis is a rare finding of ectopic splenic tissue found within the thoracic cavity, abdomen or peritoneal cavity. Most cases occur in the abdomen and the thoracic location is a comparatively rare finding. In thoracic splenosis the splenic tissue most often grows in the form of a nodule and the autotransplantation is usually caused by a previous operation and/or most commonly a penetrating or blunt trauma to the thoracoabdominal region, resulting in splenic rupture and in some cases left diaphragmatic tear. In majority of the cases the patients are asymptomatic and are incidentally diagnosed with left hemithorax pulmonary lesions found via chest radiography or thoracic computed tomography. We present a 45-year-old Caucasian male who was incidentally diagnosed with parenchymal thoracic splenosis secondary to a gunshot wound to the abdomen 13 years ago that resulted in distal pancreatectomy, splenectomy and gastrorrhaphy. In this case report we will briefly discuss the current updates in the literature regarding thoracic splenosis, and highlight the fact that the findings raise the suspicion of malignancy requiring numerous investigations yet early recognition of thoracic splenosis can prevent unnecessary tests and procedures. Preoperative diagnosis of splenosis should be made with the use of nuclear imaging studies such as the ^99m^Tc heat-damaged erythrocyte study rather than computed tomography-guided biopsy or invasive surgery.

**Keywords:**

Thoracic splenosis; Computed tomography; Ppancreatectomy; Splenectomy; Gastrorrhaphy

## Introduction

Splenosis is a rare finding of ectopic splenic tissue found within the thoracic cavity, abdomen or peritoneal cavity. Most cases occur in the abdomen and the thoracic location is a comparatively rare finding. In thoracic splenosis the splenic tissue most often grows in the form of a nodule and the autotransplantation is usually caused by a previous operation and/or most commonly a penetrating or blunt trauma to the thoracoabdominal region, resulting in splenic rupture and in some cases left diaphragmatic tear. In majority of the cases the patients are asymptomatic and are incidentally diagnosed with left hemithorax pulmonary lesions found via chest radiography or thoracic computed tomography.

## Case Report

**Figure 1.**
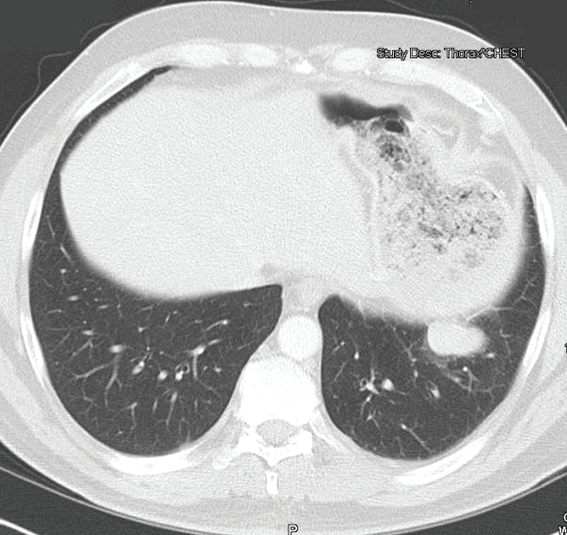
CT scan of the chest and abdomen revealed presence of a 3.4 cm mass adjacent to the left hemidiaphragm.

**Figure 2.**
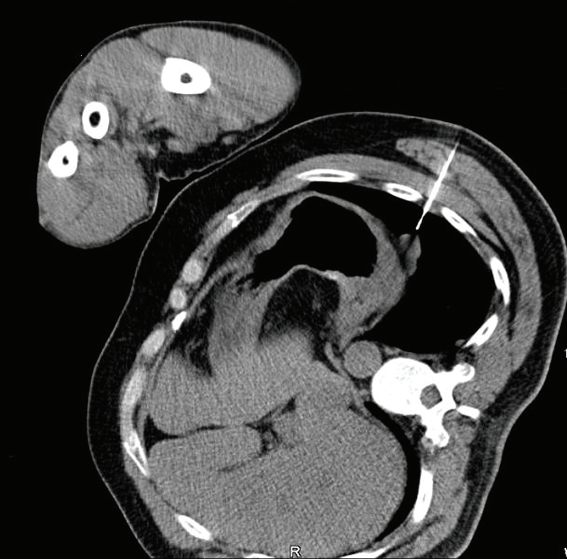
CT-guided biopsy of the mass which subsequently ruled out malignancy.

**Figure 3.**
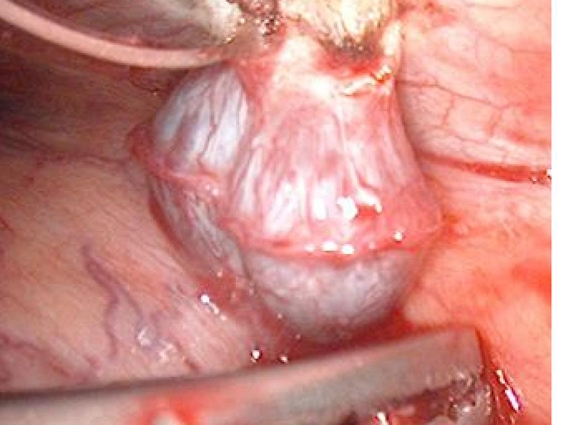
Video-assisted thoracoscopic surgery (VATS) revealed presence of the mass in the thoracic area.

A 45-year-old Caucasian male who was hospitalized for pneumonia two months prior had a follow-up chest x-ray that revealed a stellate-shaped opacity in the right lower lobe. The patient subsequently had a computed tomography (CT) scan of the chest two months after the chest x-ray that revealed no abnormalities in the right lung. However, there was a nodule in the left lower lobe that measured 3.4 cm and was located directly above and possibly contiguous with the left diaphragm. The patient denied cough, hemoptysis or weight loss. The past medical history of this patient included hypertension and hepatitis C. Surgical history included a distal pancreatectomy, splenectomy, gastrorrhaphy, and chest tube placement status post gunshot wound to the thoracoabdominal region 13 years prior (1994). The patient denied smoking, alcohol or substance abuse but was a former methamphetamine user many years ago. He has been unemployed for many years. He had no known allergies and his only medications were thiazide diuretic and an angiotensin converting enzyme inhibitor for hypertension. After incidentally discovering the 3.4 cm mass adjacent to the left hemidiaphragm (Fig. 1), the next step was a CT-guided biopsy (Fig. 2) to rule out possible malignancy. The biopsy was essentially non-diagnostic and negative for fungi or tuberculosis. The next procedure was a left video assisted thoracoscopic surgery (VATS), which was further converted to a thoracotomy with excision of left pleural mass (Fig. 3). The gross specimen appeared as brown-tan homogenous tissue fragment. Microscopically, wedge excision of the left lower lobe nodule showed lymphoid follicles with areas reminiscent of normal splenic architecture: red pulp and white pulp with surrounding areas of fibrosis. Based on the pathology of the excision and on the patient’s past history of thoracoabodominal trauma resulting in a splenectomy, the diagnosis of thoracic splenosis was made. Overall, the patient tolerated procedure well.

## Discussion

Thoracic splenosis is a manifestation of displaced splenic tissue found within the left hemithorax secondary to spleen trauma and left diaphragmatic breach. Thoracic splenosis was initially discovered in 1896 by a German physician via autopsy on a 25-year-old male [1]. Since then and to our knowledge, there have been only 38 reported cases of thoracic splenosis [2]. In nearly all of these cases the patients had no symptomatic complaints. We found only four cases where patients with ectopic thoracic splenic tissue presented symptomatically: two patients reported recurrent hemoptysis [3, 4], one patient complained of productive cough [5], and another patient had pleuritic chest pain [6]. Furthermore, thoracic splenosis was found incidentally in nearly all the reported cases, and practically all the patients had a history of thoracoabdominal trauma resulting in a splenectomy. In addition, thoracic splenosis has only been reported within the left thorax. The average period of diagnosing thoracic splenosis from the time of trauma is roughly 21 years and ranges from 6 to 46 years [2, 7]. Splenosis has been found to occur in about 65% of splenic ruptures. Of these reported cases, most were located in the abdomen and less than 20% were found within the thoracic cavity [8]. Abdominal splenosis is the most common type of splenosis, with most common sites of autotransplantation of the spleen being the mesentery, peritoneum, and omentum.

On the other hand, thoracic splenosis most commonly involves a diaphragmatic tear, or rarely a diaphragmatic hiatus, and small pieces of splenic tissue are displaced into the left hemithorax through the diaphragmatic opening(s). The most common location of thoracic splenosis occurs in the pleural cavity; but we found one case where splenic tissue had seeded into the lung parenchyma secondary to lung laceration [9]. The lung laceration in both cases was due to a chest tube insertion. Regardless of location, the ectopic splenic tissue receives its blood supply from adjacent tissue, and eventually evolves into mature tissue via a slow benign process. Thoracic splenosis should be highly suspected in patients presenting with chest x-ray or CT scan impressions of left hemidiaphragm lesion(s) including a history of spleen trauma. An even greater suspicion should be investigated if there is a history of splenectomy and/or diaphragmatic tear.

Regardless of the symptoms reported, if there is a high index of suspicion for splenosis then radionuclide scanning methods can lead to a preoperative diagnosis without the need for invasive techniques. Early recognition of these associations is extremely important and physicians must recognize the key features in order to commence appropriate imaging studies to avoid biopsy or surgery. However, some cases may be difficult to diagnose, especially if features suggesting splenosis are not recognized. In our case, thoracic splenosis and lymphoma were both among the differential diagnoses and either possibility could not be ruled out even after a CT-guided biopsy. Therefore, the patient required a thoracotomy. Diagnosis of splenosis can be made by biopsy, excision of the suspected mass, nuclear imaging, fine needle aspiration, and/or ferumoxide magnetic resonance imaging (MRI). Although there are no comparative studies that investigate the ideal procedure to diagnose splenosis, CT-guided biopsies and fine needle aspirations have returned inconclusive or misleading in multiple cases [10-12]. For instance, fine needle aspiration cytology of splenosis revealing small and medium sized lymphocytes may lead to the wrong diagnosis of lymphoproliferative disorder [13]. Less invasive investigations that may confirm the diagnosis of splenosis include the ^99m^Technetium (Tc) sulfur colloid, indium 111-labeled platelet, ^99m^Tc heat-damaged erythrocyte, or the ^99m^Tc white blood cell scan [14, 15]. Among the four nuclear scanning methods, the ^99m^Tc heatdamaged erythrocyte study has the highest specificity due to reduced uptake by the normal liver [2]. The ^99m^Tc heat-damaged erythrocyte study of the spleen and the liver uses heat-damaged erythrocytes which are localized to the spleen, where damaged erythrocytes are sequestered and phagocytosed. The indium 111-labeled platelet study is also known for high specificity and sensitivity due to splenic sequestration and phagocytosis [16]. The ^99m^Tc sulfur colloid scintigraphy is not as sensitive and specific as the ^99m^Tc heat-damaged erythrocyte study or the indium 111-labeled platelet scan [17]. Lastly, although the ^99m^Tc white blood cell scan can be used to diagnose splenosis, it is usually used for bone diseases such as osteomyelitis, as it is considered the least specific nuclear imaging study [18].

Ferumoxide MRI is another technique used to diagnose splenosis in a patient with a high index of suspicion for splenosis [19]. Ferumoxide is a superparamagnetic iron oxide, which is taken up and destroyed by the reticuloendothelial system. If multiple splenic nodules are present, there will be consistency in intensity among the nodules on MRI. MRI has shown advantages over nuclear medicine techniques by combining a higher spatial resolution with a physiological test of reticuloendothelial cell uptake [20]. Due to MRIs superior contrast resolution compared with CT or x-ray, MRI is an even more enhanced way to diagnose splenosis coupled with the history of previous throacoabdominal injury and previous splenectomy. Even though nuclear scintigraphy has been mentioned as the diagnostic method of choice [21], further research is needed because there are no studies that have directly compared nuclear imaging studies to ferumoxide MRI in diagnosing thoracic splenosis. In our case, there was no use of radionuclide scanning methods or MRI due to the preoperative differential diagnosis consisting of malignancy. Investigations to aid in the diagnosis of splenosis include peripheral blood smears. In peripheral blood smears the chronic manifestations of splenectomy are marked variation in size and shape of erythrocytes (anisocytosis, poikilocytosis) and the presence of Howell-Jolly bodies (nuclear remnants), Heinz bodies (denatured hemoglobin), basophilic stippling, and an occasional nucleated erythrocytes. When such erythrocyte abnormalities appear in a patient whose spleen has not been removed, one should suspect splenic infiltration by tumor or splenosis that has interfered with its normal culling and pitting function [22]. If those features on peripheral blood smear are acknowledged early on, then further investigation using the ^99m^Tc heat-damaged erythocyte radionuclide study may be useful in identifying ectopic splenic tissue.

Thoracic splenosis is rare but should be considered in the differential diagnosis of left-sided pleural-based pulmonary nodules. Patients with splenosis may carry a specific history of traumatic injury resulting in splenectomy and imaging studies that show healed rib fractures and/or nodules. Without this specific history, differential diagnoses should be considered. Among differentials for unilateral pleural-based nodules are lymphoma, infectious lesions, rheumatologic lesions, vascular lesions, hamartomas, neoplasms, granulomas, mucoid impaction, atelectasis, localized fibrosis, extramedullary hematopoiesis, malignant mesothelioma, thymoma, pleural metastases, or posttraumatic pleural scarring. If he patient presents with multiple nodules or masses, malignant mesothelioma and localized fibrosis may be ruled out, as they are solitary in nature [23]. If the mediastinum is not involved, then invasive thymoma is less likely [23]. Pleural lymphomas, such as Hodgkin and Non-Hodgkin Diseases are accompanied with pleural effusion. In pleural lymphomas, recurrence is usually seen and there may be an increase in the size and number of nodules within a brief time period. In contrast, thoracic splenosis is benign in nature and there are usually minimal changes in size and the number of nodules throughout the patient’s course. Intrathoracic extramedullary hematopoiesis is a rare condition and the ^99m^Tc scan is the most sensitive test to be used if suspected [23]. Unlike splenosis, intrathoracic extramedullary hematopoiesis can develop bilaterally and occurs mainly in the posterior mediastinum along the vertebrae. If there is a high index of suspicion for the diagnosis of splenosis, then a nuclear imaging scan should be ordered. If malignancy remains part of the differential diagnosis, then a VATS or thoracotomy should be performed.

Thoracic splenosis has been mainly diagnosed intraoperatively. However, excision of splenic nodules should only be considered if the patient is symptomatic or if the diagnosis is unconfirmed, such as our case where malignancy was suspected. Otherwise excision of the nodules has shown no clinical significance in asymptomatic patients and surgery may cause unnecessary complications [10-12]. Interestingly, splenosis has been postulated to have a protective role against postsplenectomy sepsis [24], however, its protective activity is not fully understood and has not been thoroughly studied. The most serious consequence of splenectomy is increased susceptibility to bacterial infections, particularly those with capsules such as Streptococcus pneumoniae, Neisseria meningitides, haemophilus influenzae, and some Gram-negative enteric organisms [25]. Some researches have determined that splenic autotransplantation enhances clearance of pneumococci from the bloodstream, increases levels of IgM, and increases opsonic activity [24]. However, it has been postulated that 50% or more of original splenic tissue is needed for protection against encapsulated microorganisms [26]. Furthermore, splenosis alone may not provide adequate defense and early prophylactic administration of penicillin and vaccination is still advised [25].

In conclusion, due to the low incidence of complications, thoracic splenosis is known as a slow growing, benign condition that is most often diagnosed incidentally. Thoracic splenosis should be highly suspected in a patient with CT or MRI of the chest showing left pleural nodule(s) in addition to having a past history of trauma to the abdomen or thorax with splenectomy and/or diaphragmatic injury. The location of thoracic splenosis most often occurs in the pleura but may be found in the parenchyma if the lung tissue was lacerated (for example, by chest tube placement). Preoperative diagnosis of splenosis in these patients should be made with the use of nuclear imaging studies such as the ^99m^Tc heat-damaged erythrocyte study rather than CT-guided biopsy or invasive surgery. Excision of the nodule is not indicated if the patient is asymptomatic, however diagnosis has frequently been made intraoperatively in order to rule out malignancy. The protective role of splenosis is controversial, although patients with splenosis have been reported to have a decreased rate of postsplenectomy sepsis, increased opsonin activity, and increased IgM activity.
